# YersiniaBase: a genomic resource and analysis platform for comparative analysis of *Yersinia*

**DOI:** 10.1186/s12859-014-0422-y

**Published:** 2015-01-16

**Authors:** Shi Yang Tan, Avirup Dutta, Nicholas S Jakubovics, Mia Yang Ang, Cheuk Chuen Siow, Naresh VR Mutha, Hamed Heydari, Wei Yee Wee, Guat Jah Wong, Siew Woh Choo

**Affiliations:** Genome Informatics Research Laboratory, High Impact Research Building (HIR) Building, University of Malaya, 50603 Kuala Lumpur, Malaysia; Department of Oral Biology and Biomedical Sciences, Faculty of Dentistry, University of Malaya, 50603 Kuala Lumpur, Malaysia; Center for Oral Health Research, School of Dental Sciences, Newcastle University, Framlington Place, Newcastle upon Tyne, United Kingdom; Faculty of Computer Science and Information Technology, University of Malaya, 50603 Kuala Lumpur, Malaysia

**Keywords:** YersiniaBase, *Yersinia*, Genomic resources, Comparative analysis

## Abstract

**Background:**

*Yersinia* is a Gram-negative bacteria that includes serious pathogens such as the *Yersinia pestis*, which causes plague, *Yersinia pseudotuberculosis*, *Yersinia enterocolitica*. The remaining species are generally considered non-pathogenic to humans, although there is evidence that at least some of these species can cause occasional infections using distinct mechanisms from the more pathogenic species. With the advances in sequencing technologies, many genomes of *Yersinia* have been sequenced. However, there is currently no specialized platform to hold the rapidly-growing *Yersinia* genomic data and to provide analysis tools particularly for comparative analyses, which are required to provide improved insights into their biology, evolution and pathogenicity.

**Description:**

To facilitate the ongoing and future research of *Yersinia*, especially those generally considered non-pathogenic species, a well-defined repository and analysis platform is needed to hold the *Yersinia* genomic data and analysis tools for the *Yersinia* research community. Hence, we have developed the YersiniaBase, a robust and user-friendly *Yersinia* resource and analysis platform for the analysis of *Yersinia* genomic data. YersiniaBase has a total of twelve species and 232 genome sequences, of which the majority are *Yersinia pestis*. In order to smooth the process of searching genomic data in a large database, we implemented an Asynchronous JavaScript and XML (AJAX)-based real-time searching system in YersiniaBase. Besides incorporating existing tools, which include JavaScript-based genome browser (JBrowse) and Basic Local Alignment Search Tool (BLAST), YersiniaBase also has in-house developed tools: (1) Pairwise Genome Comparison tool (PGC) for comparing two user-selected genomes; (2) Pathogenomics Profiling Tool (PathoProT) for comparative pathogenomics analysis of *Yersinia* genomes; (3) YersiniaTree for constructing phylogenetic tree of *Yersinia*. We ran analyses based on the tools and genomic data in YersiniaBase and the preliminary results showed differences in virulence genes found in *Yersinia pestis* and *Yersinia pseudotuberculosis* compared to other *Yersinia* species, and differences between *Yersinia enterocolitica* subsp. *enterocolitica* and *Yersinia enterocolitica* subsp. *palearctica*.

**Conclusions:**

YersiniaBase offers free access to wide range of genomic data and analysis tools for the analysis of *Yersinia*. YersiniaBase can be accessed at http://yersinia.um.edu.my.

**Electronic supplementary material:**

The online version of this article (doi:10.1186/s12859-014-0422-y) contains supplementary material, which is available to authorized users.

## Background

*Yersinia* is a genus of Gram-negative rod shaped bacteria belonging to the Enterobacteriaceae family. The majority of *Yersinia* species are environmental and non-disease causing in mammals and can be isolated from many locations such as fresh water and soil. Some species can also be isolated from sources like patient’s body or even dog faeces [1-4]. Three of the *Yersinia* species are known to cause disease in human: the plague bacillus *Y. pestis* and the enteropathogens *Y. enterocolitica* and *Y. pseudotuberculosis*. The most notorious and virulent species in this genus, *Y. pestis*, the causative agent of plague, is a recently emerged lineage from *Y. pseudotuberculosis* [3]. Plague, which has caused three pandemics in human history [5,6], is mainly a zoonotic infection caused by *Y. pestis*, a rodent pathogen that can be transmitted to humans through the bite of an infected flea. Infection can lead to acute febrile lymphadenitis also called bubonic plague or may cause septicemia, pharyngeal, meningeal and fatal pneumonia plague [7]. The World Health Organization (WHO) has categorized this disease as re-emergent in some parts of the world as plague reappeared in Malawi, Mozambique, and India in 1994, in Algeria in 2003, and in Libya in 2009 [7-12]. The potential re-emergence of plague is a significant worldwide public health hazard. The problem has become even more severe with the emergence of multi-drug resistant strains and the potential use of plague as a bioweapon [13-15].

*Yersinia enterocolitica*, the other notorious human pathogen of the genus *Yersinia* is a zoonotic agent that causes gastrointestinal disease in humans. This species is also responsible for causing reactive arthritis and erythema nodosum. Enteropathogenic *Yersinia*, the etiological agents for yersiniosis, are generally acquired by consuming raw or inadequately thermally processed pork or milk as well as vegetable products and ready-to-eat meals [16,17]. As per the published annual e-reports of the European Food Safety Authority (EFSA) on the prevalence of zoonoses and foodborne pathogens in 2012, yersiniosis was listed as the third-most-common enteropathogenic disease after campylobacteriosis and salmonellosis [18].

All the three human pathogens share the similarity of carrying an extrachromosomal 70-kb pYV virulence plasmid that encodes the Ysc type III secretion system (TTSS), the protein microinjection apparatus and *Yersinia* outer proteins (Yops), a set of translocated effector proteins [19]. The TTSS is highly conserved among Gram-negative pathogens and is used by *Yersinia* pathogens to inject six different Yop effectors into the host cell [20]. These Yop effectors allow *Yersinia* to resist phagocytosis and also interfere with signalling pathways within the host cell [19].

Although the three pathogenic *Yersinia* species have been extensively studied for many years, the other *Yersinia* species, which include *Y. frederiksenii, Y. intermedia, Y. kristensenii, Y. bercovieri, Y. massiliensis, Y. mollaretii, Y. rohdei, Y. ruckeri,* and *Y. aldovae* have remained largely ignored and are not well studied especially at the genome level. These species are isolated from the environment as well as from infected humans and do not possess typical *Yersinia* virulence markers. Formerly these species were grouped under *Y. enterocolitica* as different biogroups and they have been considered as *Y. enterocolitica*-like species in some papers although they are clearly distinct from *Y. enterolitica* [2,21]. Although this group of species is generally considered non-pathogenic, some strains have been associated with infections in mankind [21,22]. Therefore, it is important for researchers to analyse them further and to determine whether they have the potential to become pathogenic, since the acquisition of virulence genes through horizontal gene transfer is very common in prokaryotes [23].

With *Y. pestis* being already declared as re-emergent, *Y. enterocolitica* being listed as the third-most-common enteropathogen and several ‘non-pathogenic’ strains of *Yersinia* showing signs of infections in humans, it is clear that the genus *Yersinia* is in a state of evolution and further study is required not only on its pathogenic strains but also on the non-pathogenic stains. In recent years, a new trend of collecting bacterial genomes into a single database has emerged as an effective way to analyse their genomes. Consequently many specialized genomic databases have been developed, especially for human disease pathogens. There are a number of databases, such the Microbial Genome Database for Comparative Analysis (MBGD) [24-26] and the Integrated Microbial Genomes (IMG) [27-30] system, which provide a wide array of microbial genomes including some *Yersinia* strains for comparative genomics. Another such database worth mentioning is PATRIC [31], which offers genomic and virulence factors information of some of the *Yersinia* strains, however does not provide functionalities for comparing, clustering and visualizing the virulence gene profiles of the user-selected *Yersinia* strains. These databases do not provide the option for comparative pathogenomics analysis based on the virulence factors of the strains. Moreover, most of the existing biological databases lack user-friendly web interfaces which allows real-time and fast querying and browsing of genomic data. In order to provide researchers with a specialized platform to access the genomic data of the pathogenic and the generally considered non-pathogenic *Yersinia* species, we have developed the genus specific database, YersiniaBase. YersiniaBase stores the *Yersinia* genomic resources along with various analysis tools particularly for comparative analysis of *Yersinia* strains. One of the features of this database is the PathoProT which has been developed in-house, with the intention of identifying the potential virulence markers in the *Yersinia* genus. This comparative analysis platform will help users gain a deeper insight into the different species of *Yersinia* especially in the area of their pathogenicity. Using the resources and the tools provided in YersiniaBase, we have carried out genomic analyses on several pathogenic and non-pathogenic strains of *Yersinia*. The preliminary results shown in this paper not only helped to identify some of the genome characteristics of *Yersinia* but also demonstrated the suitability and the usefulness of the database and the tools hosted by it for *Yersinia* research.

## Construction and content

### Genome sequences

A total of 232 genome sequences of *Yersinia*, which include both draft and complete genomes in FASTA format were downloaded from National Center for Biotechnology Information (NCBI) [32]. Approximately 90% of the downloaded genome sequences belong to *Y. pestis*. All generally considered non-pathogenic *Yersinia* species were only available as draft genomes. A list of draft and complete genomes for each *Yersinia* species is shown in Table 1.

**Table 1:** **Number of draft and complete genomes of each** ***Yersinia*** **species in YersiniaBase**

**Species**	**Number of draft genomes**	**Number of complete genomes**
*Y. aldovae*	1	0
*Y. bercovieri*	1	0
*Y. enterocolitica*	8	3
*Y. frederiksenii*	1	0
*Y. intermedia*	1	0
*Y. kristensennii*	1	0
*Y. massiliensis*	1	0
*Y. mollaretii*	1	0
*Y. pestis*	196	12
*Y. pseudotuberculosis*	0	4
*Y. rohdei*	1	0
*Y. ruckeri*	1	0

Majority of the strains belonged to *Yersinia pestis.*

### Annotation of genome sequences

For consistency, all of the downloaded genome sequences were annotated by using Rapid Annotation using Subsystem Technology (RAST) [33]. RAST can predict open reading frame (ORF), function of the ORF, nucleotide sequence and amino acid sequence in user-submitted genome. Besides RAST, PSORTb version 3.0 [34] was used to determine the subcellular localization of the proteins predicted by the RAST engine. We also computed the hydrophobicity and molecular weight of the RAST-predicted proteins using in-house developed Perl scripts. The summary of genome annotations of each strain is shown in Additional file 1: Table S1.

### Database and web interface design

We implemented a relational database using MySQL version 14.12 (http://www.mysql.com). All of the biological data of *Yersinia* strains were rearranged to fit into the designed database schema and stored in the MySQL database (Additional file 2: Figure S1).

A web interface was built by using HyperText Markup Language (HTML), HyperText Preprocessor (PHP), JavaScript, jQuery, Cascading Style Sheets (CSS) and AJAX. CodeIgniter version 2.1.3, a popular PHP framework was used in order to a provide model-view-controller (MVC), which can separate application data, presentation and background logic and process into three distinct modules. This allowed the source codes and *Yersinia* biological data to be arranged in a clear and organized manner, indirectly allowing easier future updating of YersiniaBase.

### Bioinformatics tools

We developed several bioinformatics tools and integrated them into YersiniaBase. We used Python, Perl, BioPerl [35] and R languages to develop the PGC for comparing between two genomes through global alignment, PathoProT for generating heat map to visualize presence and absence of virulence genes in selected genomes and YersiniaTree to generate phylogenetic tree of the *Yersinia* strains based on their housekeeping genes and 16S rRNA. The use of these three popular scripting languages allowed us to create complex pipelines, that perform back-end calculations, aided communications between the web server and the application server and also easier transfer of data from the web server to the application server and vice versa.

## Utility and discussion

### Overview of the database pipeline

All the *Yersinia* genomes after being downloaded from National Center for Biotechnology Information (NCBI) website (http://www.ncbi.nlm.nih.gov) were annotated using the Rapid Annotation using Subsystem Technology (RAST) server [33] for consistency. All the CDS, ORF and RNA sequences predicted by the RAST server along with their annotations and predicted protein functions were downloaded. Then using the PSORTb version 3.0 [34] the subcellular localization of the RAST predicted proteins were determined. Further analyses of the protein-coding genes were performed using Bio-Perl [35] and also in-house Perl script to obtain information such as calculation of GC content (%), predicted hydrophobicity (pH), and molecular weight (Da) of the encoded proteins. All this information was then stored in the MySQL database server.

### Web interface and functionalities of YersiniaBase

On entering the home page of YersiniaBase, visitors can view the news & conferences, blogs & information and the most recent published papers that are related to *Yersinia*, which we manually compile from various sources. The “Browse” menu allows the visitors to view the list of *Yersinia* species currently available in YersiniaBase, with each “View Strains” button leading the visitors to the “Browse Strains” page, displaying all available strains of that respective species. In the “Browse Strains” page a general description of that particular species is given along with a table listing the strains of that species. Each strain is linked to their corresponding taxonomic classification page in NCBI and also to their page in Genome Online Database (GOLD). Furthermore, by clicking on the “Details” icon, visitors can obtain more comprehensive information of that particular strain such as their source and time of isolation, which we retrieved from NCBI, along with the list of ORFs, their respective function, start and stop positions in a tabular fashion in the “Browse ORF” page. Apart from that, each ORF is linked to its corresponding UniProt page along with their ORF ID being linked to its corresponding page in NCBI. By clicking on the Contig ID of each ORF, the corresponding contig information available in NCBI can be accessed. The Details button of each ORF leads the visitor to the “ORF Detail” page displaying the detailed information of that ORF such their type, start and stop positions, lengths of nucleotide as well as amino acid sequences. It further provides information on functional classification, subsystem (if available), strand, subcellular localization, hydrophobicity (pH) and molecular weight (Da). The page also displays the amino acid and the nucleotide sequence of the ORF along with the Genome Browser. The “Genome Browser” menu links user to JBrowse [36] in YersiniaBase. JBrowse allows users to view the position of each ORF at each genome graphically.

We also integrated in-house tools and as well as other tools into YersiniaBase to add functionalities to this *Yersinia* research platform. The “Tools” menu allows user to perform a BLAST search [37] against the *Yersinia* strains curated in YersiniaBase, as well as an exclusive BLAST search against the Virulence Factor Database (VFDB) [38-40]. Besides the BLAST search, users can also perform pairwise alignment of any two *Yersinia* genomes present in YersiniaBase of their choice by using the PGC, draw a heat map of the virulence genes profiles by using the PathoProT or construct phylogenetic tree by using YersiniaTree.

“Search” menu allows user to search the functional classification of a specific species and strain by providing keyword or ORF ID. Besides performing searching, user can also download the genome sequence, ORF annotation details in table format, ORF sequence, ribonucleic acid (RNA) and coding sequence (CDS) through “Download” menu. The overview of the functionalities of YersiniaBase is shown in Additional file 3: Figure S2.

### Browsing *Yersinia* strains

The browse page of this database summarizes a list of 12 *Yersinia* spp., and their respective number of draft genomes and complete, as shown in Table 1. The “View Strains” button on the right of each species leads the user to a list of strains of the chosen species. In the table, the information available to the user are strain status (draft genome or complete genome), genome size in mega base pair (Mbp), percentage of GC content (%), number of contigs, number of predicted CDS, number of predicted tRNA and number of predicted rRNA. On the right of each strain, the user can find a small icon, which provides a hyperlink enabling the user to find a list of predicted ORFs of the selected strain. From there, the user can see the ORF type (CDS or RNA), functional classification, contig, start position and stop position associated with each predicted ORF of the strain. The small icon on the right of each ORF brings the user to another page which allows them to see additional information of the predicted ORF besides that described above, which includes nucleotide length (bp) and the sequence, predicted polypeptide length (amino acids) and the direction of transcription, subcellular localization, hydrophobicity (pH), molecular weight (Da) and SEED subsystem. The page is also equipped with JBrowse a fast and modern JavaScript-based genome browser [36] which will enable the user to navigate genome annotations and visualize the location of the ORF of the selected *Yersinia* strain. On the top of the page, the user can find a “Download” button to download annotation details, amino acid sequence and nucleotide sequence of the predicted ORF.

### Advanced real-time searching

YersiniaBase stores large amounts of data related to *Yersinia*, most of them being ORFs which were predicted from the RAST engine, with more than one million genes and coding sequences. Hence, it is impractical to browse for the required information page by page as this will greatly slow down the progress. In order to solve the problem of searching for specific genes or functional classifications in such a large database, we implemented a real-time search engine using AJAX in YersiniaBase. We designed the real-time search engine in a way such that the communications between web interface and MySQL database is asynchronous; refreshing of web page is not needed to display the list of suggested functional classifications that match the entered keyword. As soon as the user enters one keyword, the real-time search engine will retrieve a list of functional classifications that contain the keyword entered by the user and display it to the user seamlessly. Once the user selects one item from the list, they will be presented with a table which contains a list of genes that are related to the entered keyword.

### In-house developed pairwise genome comparison tool (PGC)

As pointed out earlier, *Y. pestis* has already been declared as re-emergent and *Y. enterocolitica* is listed as the third-most-common enteropathogen. Many of the previously considered non-pathogenic strains of *Yersinia* have also shown signs of infections in humans. It is evident that the genus *Yersinia* is constantly evolving and further study is required not only of the pathogenic strains but also of the non-pathogenic stains to gain a clear understanding of its biology. To have a clear idea of the lifestyle, the evolution and an extended view of the gene pool of a species, genomic information from a single *Yersinia* genome is unlikely to be sufficient. For detailed insights into the variations between different strains at the genetic level, the evolutionary changes among *Yersinia* species, the orthologous genes in species, as well as the genes that give each organism its unique characteristics, especially the potential regions associated with pathogenicity, a comparative study of multiple *Yersinia* genomes is required.

With that in mind, the PGC was developed and incorporated into YersiniaBase. This in-house developed online comparative genome analysis tool will allow the user to compare two selected *Yersinia* genomes. Unlike conventional methods which display alignments in a linear form, the PGC displays the alignments in a circular layout. On entering the web interface of PGC in YersiniaBase, the user can choose two *Yersinia* genomes of interest from the list for comparison. Alternatively, the user can upload their *Yersinia* genome sequence for comparison with the *Yersinia* genomes in YersiniaBase. Three parameters, which are minimum percent identity (%), merge threshold (bp) and link threshold (bp) can be defined manually by the user. Link threshold (LT) removes the links according to user-defined value, while merge threshold (MT) allows merging of links based on user-defined value. Link is where the region of query genome maps to region to reference genome. Merged means the merging of two links which is separated by gap into one wider link. Mapped region of query genome to reference genome will be shown if the region is higher than the link threshold; while gap will be displayed if it is higher than the merged threshold, else the links beside the gap will be merged into a wider link. If user set the merge threshold to 0Kbp, and link threshold to 1Kbp, then the result of PGC will be same as shown in Additional file 4: Figure S3(A) whereas if the merge threshold was changed to 2Kbp, the result will be same as Additional file 4: Figure S3(B).

In the PGC pipeline, after the sequences are aligned through NUCmer, the alignment results are immediately parsed to Circos [41], which then generates a circular ideogram layout to show the relationship between pairs of positions, with karyotypes and links encoding the position, size and orientation of the related genomic elements. Perl scripts were used to automate the multi-step process of this pipeline. The results generated by the PGC tool, both the NUCmer alignment results and the Circos plot can be downloaded using the “Download” button in the PGC result page. Additional file 5: Figure S4 briefly illustrates the work flow of PGC tools, describing the integration of both MUMmer and Circos, generating the required input files for the PGC to function.

At the time of writing this paper, a similar tool named Circoletto [42] already existed, which aligns two genomes by using BLAST, however PGC aligns two genomes by using NUCmer package in MUMmer 3.0 [43]. In comparison, the latter is more favourable and more suitable for whole-genome comparison as NUCmer uses global alignment which is more suitable for large-scale and rapid pairwise alignment between two large genomes while Circoletto uses BLAST, a local alignment program. PGC provides a user-friendly interface and requires no prior programming knowledge. PGC allows the user to adjust parameters such as minimum percent genome identity (%), merging of links/ribbons according to merge threshold and also the removal of links according to the user-defined link threshold through the provided online form. A histogram track showing the percentage of mapped regions along the genomes is added in the circular layout generated by PGC, making it helpful for the users to identify putative indels and repetitive regions in the compared genomes.

### Pathogenomics profiling tool (PathoProT) for comparative pathogenomics analysis

Pathogenesis is due to the presence of virulence genes in bacteria [44], which is responsible for causing disease in the host. The acquisition of individual virulence factors may convert non-pathogenic bacteria into pathogens [45]. With the availability of genome sequences of different *Yersinia* species it is essential to do comparative analyses of the virulence factors in the *Yersinia* pathogen genomes to identify new potential virulence markers and to provide new insights into pathogenicity of this genus. In order to identify potential virulence genes in the *Yersinia* strains and to facilitate comparative analyses between different *Yersinia* strains, we have developed PathoProT, a unique comparative pathogenomics analysis tool to help users to get a deeper insight into the different species of *Yersinia* especially in an area of pathogenicity.

PathoProT was designed using in-house developed Perl and R scripts, where Perl handles the initial processes while R is used to generate hierarchical clustering and heat map visualization of multiple virulence gene profiles. The user can select a list of *Yersinia* strains for comparative analysis and set the cut-off for sequence identity and completeness through the online form on the PathoProT main page. PathoProT predicts virulence genes based on sequence homology of all protein sequences of user-selected strains against VFDB [38-40] using the well-established BLAST (Stand-alone) tool of NCBI [37,46-48], which is downloaded from NCBI and embedded in the PathoProT pipeline. The virulence factors retrieved from VFDB, a database consisting of the experimentally verified virulence factors from different pathogenic organisms (VFDB Version 2012 containing a total of 19775 proteins), for PathoProT are considered as the “known virulence factors”. In the PathoProT pipeline, a BLAST search is performed with the default parameters of 50% sequence identity and 50% sequence completeness to identify the orthologs of these known virulence factors in the *Yersinia* genomes present in the YersiniaBase. However the user can change these default parameters for the BLAST search depending on their desired levels of stringency. In-house developed Perl script filters the BLAST results generated from a BLAST search against VFDB based on user-defined cut-off values for sequence identity and completeness to identify the virulence genes and selects only the user desired strains. Results from the filtering process will be used to tabulate the data matrix (strains versus virulence genes) This is followed by executing R scripts, to read the generated data matrix and perform hierarchical clustering (complete-linkage algorithm) of the *Yersinia* strains based on their virulence gene profiles. Finally, for the visualization of the virulence gene profiles, ‘pheatmap’ package is used to generate the heat map image which is the end-product of PathoProT. The use of heat map in PathoProT for visualization enables users to have a bird’s eye view of the data in a graphical representation [49], allowing users to identify and visualize the virulence gene profile in their strains of interest, and enabling comparative analyses of virulence factors among different *Yersinia* strains. Additional file 6: Figure S5 shows the flow chart of PathoProT, briefly describing the pipeline which integrates both Perl script and R script, and the processes before generating the output file.

### YersiniaTree: constructing *Yersenia* phylogenetic tree

Phylogenetic trees are important in studying the evolutionary relationships between different species, and their common ancestor. In YersiniaBase, we have designed and incorporated an automated pipeline written in Perl, YersiniaTree that will enable user to generate phylogenetic trees of the *Yersinia* strains based on their housekeeping genes and 16S rRNA. Our automated pipeline YersiniaTree, primarily requires two inputs from the user: (1) gene marker used to construct the phylogenetic tree (2) list of genomes in YersiniaBase which to be included in the tree. The automated pipeline also offers an optional feature where the users can input their nucleotide sequence in FASTA format along with the sequences which are retrieved from the database. Currently, this pipeline in YersiniaBase, allows users to choose from one out of five gene markers, which can be seen from the drop-down menu to construct the phylogenetic tree. Besides the common 16S rRNA, users can choose from any one of the following *gyr*B, *hsp*60, *rpo*B, or *sodA* for the construction of the phylogenetic tree. Previous studies have shown that phylogenetic trees based on these four housekeeping genes are more consistent with the biochemical profiles of *Yersinia* species than those based on the 16S rRNA [4]. After user provides all the necessary inputs, the front end PHP executes the backend Perl pipeline. The first step of our automated pipeline is to select target gene’s nucleotide sequence of genomes chosen by user from FASTA file where complete listings of sequences are stored, into a temporary FASTA file. The Perl script then executes MAFFT [50], which is used to perform multiple sequence alignment across nucleotide sequences. Next, the output file from MAFFT is sent to FastTree [51] to construct phylogenetic tree in Newick format, followed by visualizing of the tree in SVG format by using Newick Utilities [52]. Finally, Perl script sends out the final image to PHP to display the phylogenetic tree in web browser.

### *Yersinia* genome, gene and virulence factors database

Besides the in-house developed tools, we have also integrated BLAST as well as VFDB-BLAST into YersiniaBase. This will allow the users to perform their similarity search of their query sequences against *Yersinia* genome sequences, gene sequences and also against the virulence factors (downloaded from VFDB [38-40]) by using BLASTN, BLASTP or BLASTX. This specialized database for *Yersinia* provides faster searching against *Yersinia* sequences compared to NCBI NT or NR databases when the user is trying to find the closest *Yersinia* strains to their own query sequence.

### Features of strains in YersiniaBase

Generally, the size of the *Yersinia* genomes was observed to be between 4.3 Mbp to 4.8 Mbp. However, there were a few exceptions. For example, the genome size of *Y. ruckeri* ATCC29473, a fish pathogen, was approximately 3.7 Mbp, which is smaller than the normal range. *Y. kristensennii* ATCC33638 and *Y. massiliensis* CCUG53443 had genome sizes larger than 5Mbp. RAST annotations of all the download *Yersinia* genomes, predicted a total of 1,009,094 ORFs. 997,893 of the ORFs encoded protein while 11,201 encoded functional RNA. The subcellular localization analysis of the RAST predicted proteins using PSORTb version 3.0, showed that the major portion of the predicted proteins produced by *Yersinia* were predicted to be located in cytoplasmic region. The results obtained from PSORTb are tabulated in Table 2.

**Table 2:** **The breakdown of subcellular compartments of predicted** ***Yersinia*** **proteins and their numbers in percentage format**

**Subcellular compartments**	**Percentage of localization (%)**
Cytoplasmic	37.31
Cytoplasmic membrane	23.36
Extracellular	1.66
Outer membrane	2.52
Periplasmic	3.36
Unknown	31.80

### Comparative genomic analysis

**Figure 1:**
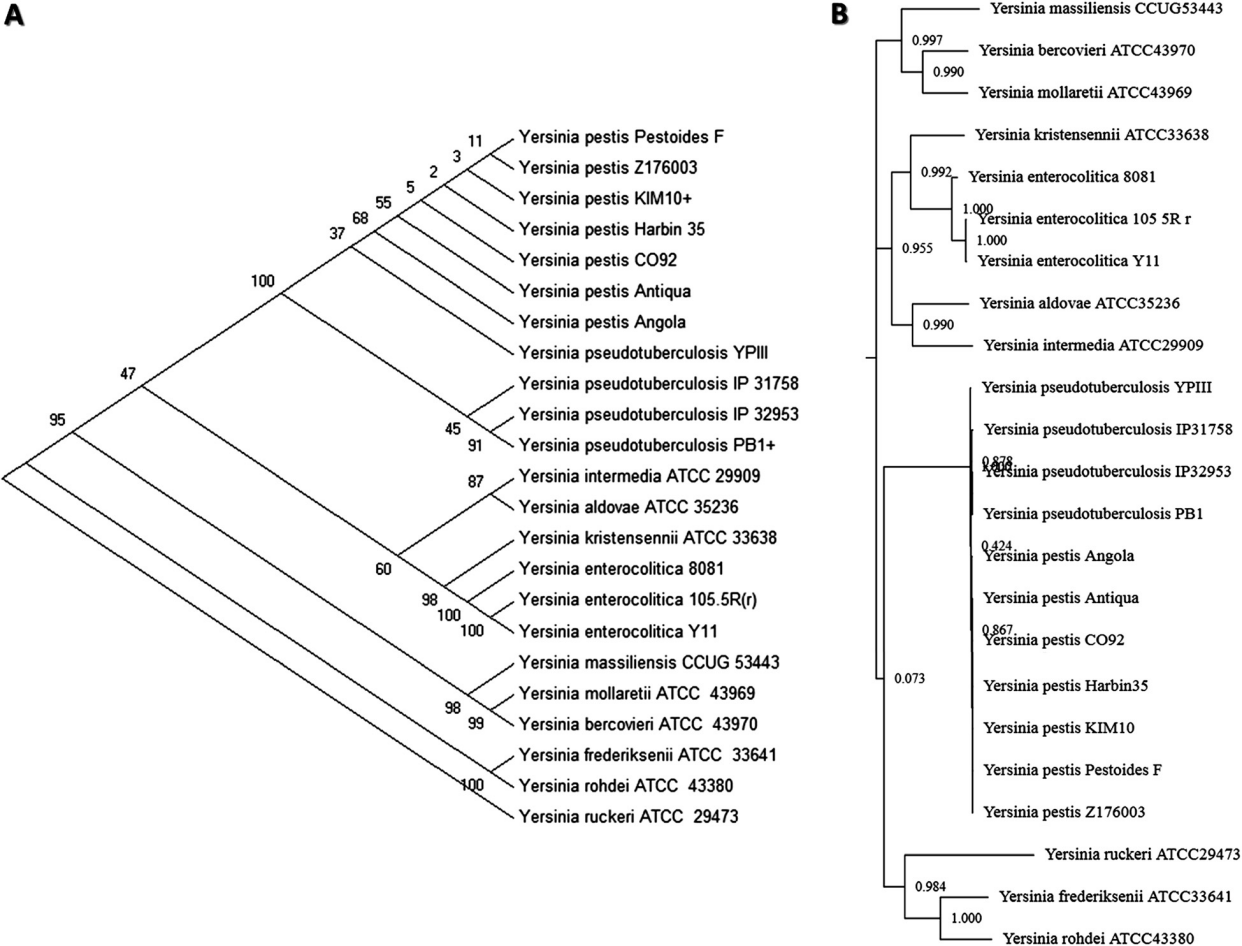
**Comparison of phylogenetic trees constructed from gyrB gene sequences. (A)** *gyr*B–based tree constructed using MEGA6. **(B)** *gyr*B–based tree constructed using in-house developed YersiniaTree.

To demonstrate the usefulness of YersiniaBase, we carried out genomic analyses of 23 *Yersinia* strains available in YersiniaBase. Three phylogenetic trees were constructed, one using the MEGA6 [53], the second one using the phylogenetic tool of YersiniaBase, YersiniaTree and the other using the in-house developed tool PathoProT. We used the neighbour-joining method, Tamura-Nei model with 1,000 bootstrap replications to build phylogenetic tree with MEGA6 [53] based on the *gyr*B gene (Figure 1A). The tree generated by YersiniaTree was also based on *gyr*B (Figure 1B). The *gyr*B gene instead of 16S rRNA gene was used for phylogenetic analysis as 16S rRNA cannot resolve the phylogenetic relationships between closely related *Yersinia* species. Housekeeping genes such as *gyrB* produce phylogenetic trees which closely correlate with the biochemical species designations. Moreover studies have shown that *gyr*B is a better phylogenetic marker than 16S rRNA in the Enterobacteriaceae [4,54-56]. The other tree was constructed using the in-house developed tool PathoProT, with hierarchical clustering (complete-linkage) algorithm based on the virulence gene profiles of the selected *Yersinia* strains. The parameters for PathoProT were set to 50 for sequence identity (%) and 50 for sequence completeness (%). We also used the in-house developed PGC Tool for finding the differences between subspecies of *Y. enterocolitica*. The parameters for the PGC Tool were set to 90 for minimum percent identity, 2,000 for link threshold (bp) and 2,000 for merge threshold (bp). The list of *Yersinia* strains selected for the analysis is shown in Table 3.

**Table 3:** **List of** ***Yersinia*** **strains used in the analysis**

**Species name**	**Strain name**
*Y. aldovae*	ATCC35236
*Y. bercovieri*	ATCC43970
*Y. frederiksenii*	ATCC33641
*Y. intermedia*	ATCC29909
*Y. kristensennii*	ATCC33638
*Y. massiliensis*	CCUG53443
*Y. mollaretii*	ATCC43969
*Y. rohdei*	ATCC43380
*Y. ruckeri*	ATCC29473
*Y. enterocolitica*	105.5R(r)
*Y. enterocolitica*	8081
*Y. enterocolitica*	Y11
*Y. pestis*	Angola
*Y. pestis*	Antiqua
*Y. pestis*	CO92
*Y. pestis*	Harbin35
*Y. pestis*	KIM10
*Y. pestis*	Pestoides F
*Y. pestis*	Z176003
*Y. pseudotuberculosis*	IP 31758
*Y. pseudotuberculosis*	IP 32953
*Y. pseudotuberculosis*	PB1+
*Y. pseudotuberculosis*	YPIII

### Phylogeny of *Yersinia* based on *gyrB*

In this paper, we constructed two phylogenetic trees based on *gyrB*, one using MEGA6 (Figure 1A) while the other using our in-house developed tool YersiniaTree (Figure 1B). The tree generated by YersiniaTree was found to be consistent with that of the tree generated by the well-established tool MEGA. Both the trees showed similarity in the clustering pattern. In both the cases the strains of *Y. pestis* and *Y. pseudotuberculosis* grouped very close each other whereas *Y. enterocolitica* formed a separate cluster. In case of *Y. enterocolitica* strains 105.5R(r) and Y11 (both of which belong to the subspecies *palearctica*) grouped together while 8081 (subspecies *enterocolitica*) separated to form a different branch in the tree generated by YersiniaTree (Figure 1B). Similar type of clustering was also observed in the tree by MEGA (Figure 1A).

### Evolution of *Y. pestis* and *Y. pseudotuberculosis*

**Figure 2:**
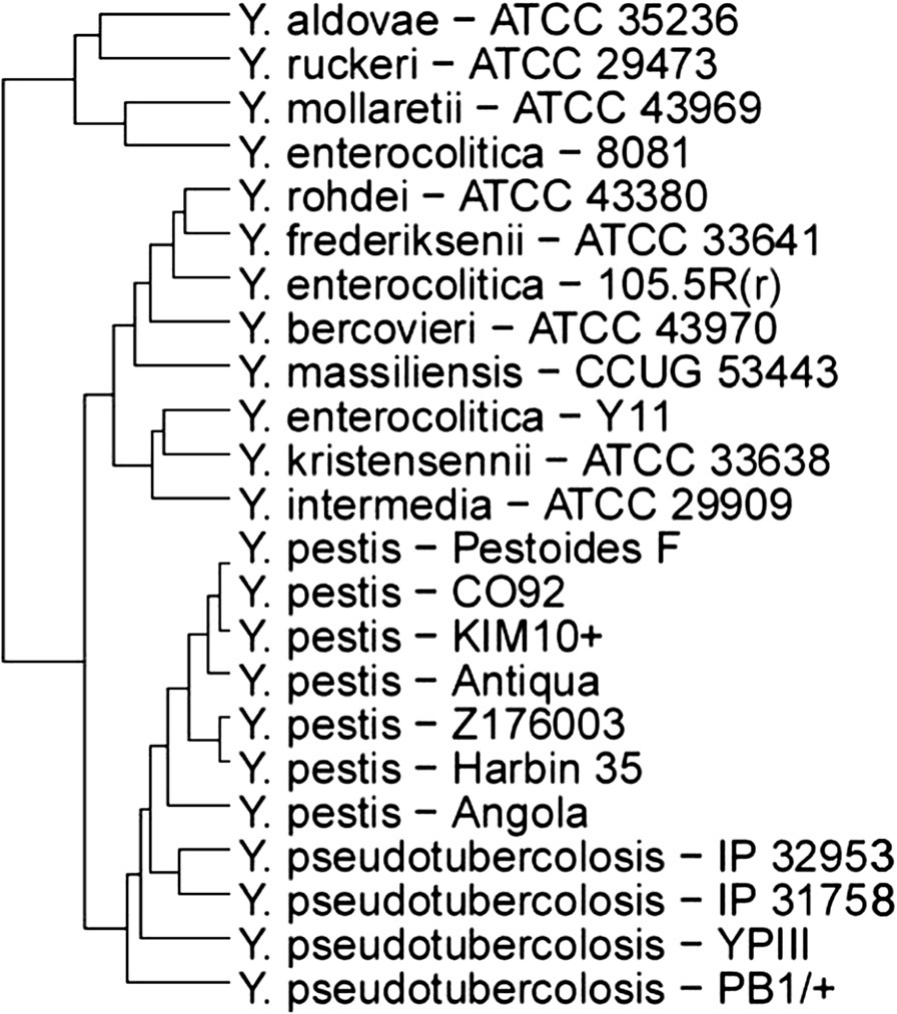
**Phylogenetic tree constructed by from virulence gene profiles with PathoProT.**

Apart from the trees generated based on *gyr*B a separate tree was constructed based on the virulence profiles of *Y. pestis* and *Y. pseudotuberculosis* strains using the PathoProT (Figure 2). Even in the tree by PathoProT (Figure 2), we found that *Y. pestis* and *Y. pseudotuberculosis* formed a cluster that was clearly separated from *Y. enterocolitica* and non-pathogenic *Yersinia* which was similar to that observed in both the trees based on *gyr*B. This similarity in the clustering pattern, suggests that the housekeeping genes and virulence genes of *Y. pestis* and *Y. pseudotuberculosis* evolved in a similar pathway and are distinct from the other *Yersinia* species. These results were in agreement with a recently published report suggesting that *Y. pestis* and *Y. pseudotuberculosis* are close relatives, however *Y. enterocolitica* shows diametric separation with most environmental species occupying intermediate branching positions between these two clusters [57,58]. The tree also indicates that *Y. pestis* Angola is the first among the *Y. pestis* strains to branch out from the closest common ancestor *Y. pseudotuberculosis* YPIII. This observation is supported by a recently published report, which stated that *Y. pestis* Angola belongs to one of the most ancient *Y. pestis* lineages sequenced to date, possessing genome characteristics which are intermediate between *Y. pseudotuberculosis* and the modern day *Y. pestis* [59]. These data suggest that *Y. pestis* is highly similar to *Y. pseudotuberculosis* genetically and the virulence genes that were lost or acquired did not cause it to diverge far from its ancestor. However, *Y. pestis* has undergone gene reduction and has accumulated pseudogenes since its emergence from *Y. pseudotuberculosis* [60,61].

### Virulence genes in *Yersinia*

**Figure 3:**
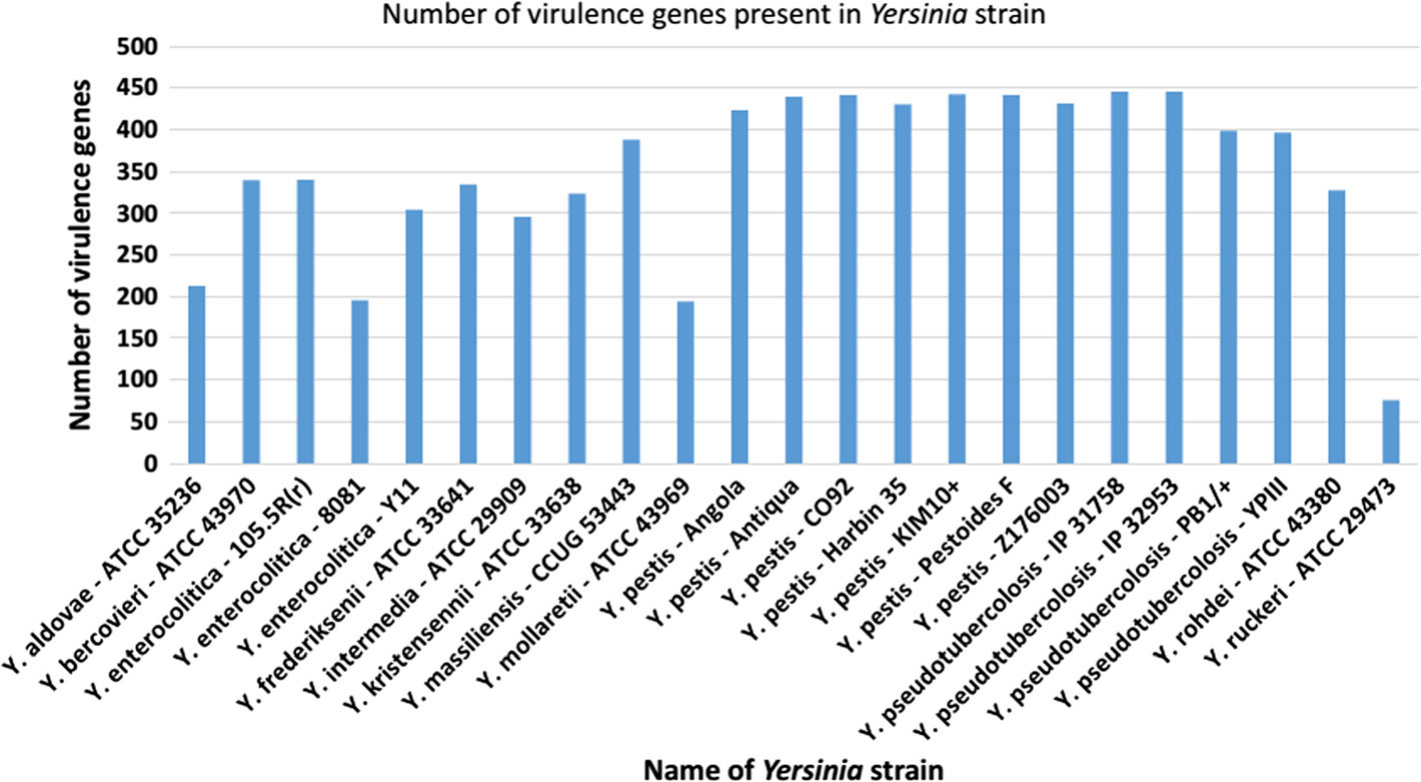
**Number of virulence genes present in each** ***Yersinia*** **strain.**

By calculating the number of virulence genes presents in every strain, we found that *Y. pestis* and *Y. pseudotuberculosis* generally have more virulence genes than other species and the number of virulence genes in different strains is more consistent within these species compared to *Y. enterocolitica* and non-pathogenic *Yersinia* (Figure 3). The number of virulence genes in non-pathogenic *Yersinia* varies from as low as 76 in *Y. ruckeri* ATCC29473, the fish pathogen to 389 in *Y. massiliensis* CCUG53443. However, it must be taken into consideration that all of the non-pathogenic *Yersinia* are only available in draft genomes and it is possible that some virulence genes have not been included in the sequences. Although *Y. enterocolitica* 8081 is a highly pathogenic strain [62], it has the third lowest number of virulence genes among *Y. enterocolitica* and non-pathogenic *Yersinia*. This suggests the other *Y. enterocolitica* and non-pathogenic *Yersinia* might only acquire virulence genes which do not play vital role in pathogenicity or the virulence genes were deactivated.

Another interesting observation from the study of the virulence genes of the *Yersinia* genomes is that *Y. pestis* and *Y. pseudotuberculosis*, which formed a cluster in both the trees, also had a consistency in the number of virulence genes present in their genomes, with an average of 436 and 422 identified virulence genes in case of *Y. pestis* strains and *Y. pseudotuberculosis* stains respectively, while on the other hand *Y. enterocolitica* had an average of 280 identified virulence genes. The average number of virulence genes from *Y. enterocolitica* was significantly lower as compared to the other two pathogens in *Yersinia*. This difference in the number of virulence genes in the three pathogenic species of *Yersinia* also indicates that *Y. pestis* and *Y. enterocolitica* are close relatives and might have followed a similar evolution pathway in acquiring or losing virulence genes whereas *Y. enterocolitica* clearly followed a different evolutionary path, independent from the other two. Surprisingly, *Y. enterocolitica* 8081, which is the highly virulent biotype, did not form the same cluster with the other two strains of *Y. enterocolitica*, but rather with 4 non-pathogenic *Yersinia*.

### Absence of yersiniabactin in *Y. enterocolitica* subsp. *palearctica*

**Figure 4:**
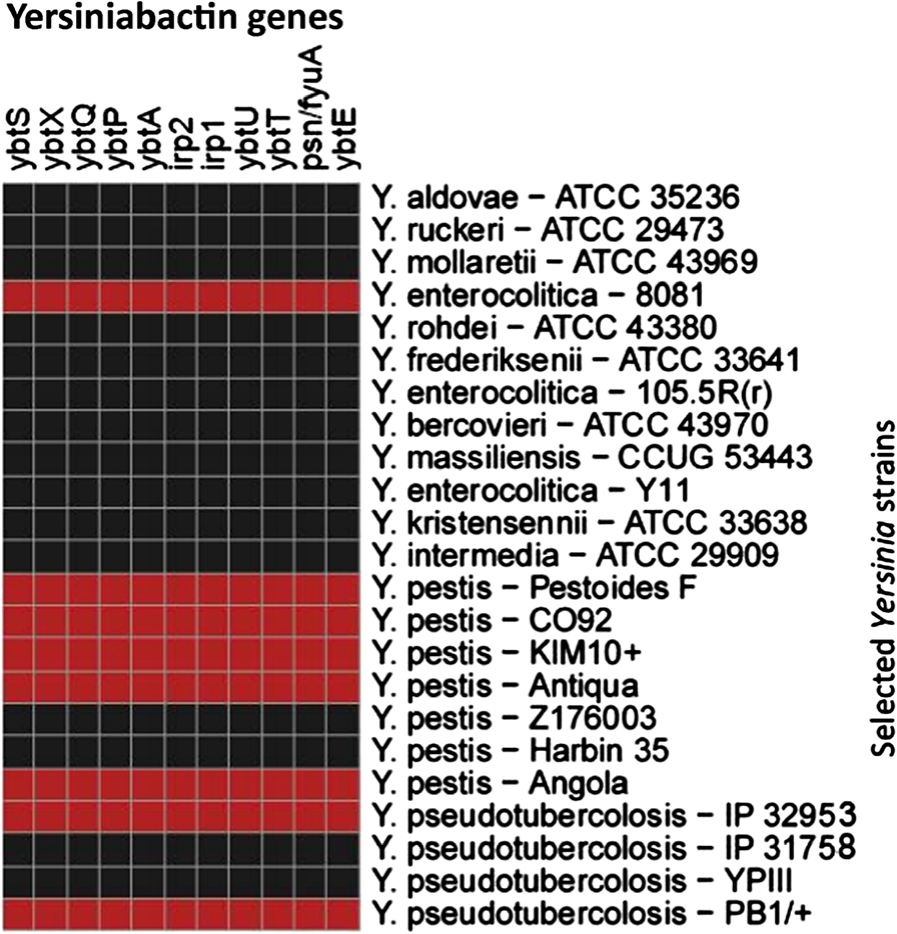
**A heat map from PathoProT showed the presence (red color) and absence (black color) of yersiniabactin genes in selected** ***Yersinia*** **genomes.** 8081 which belongs to *Y. enterocolitica* subsp. *enterocolitica* has yersiniabactin genes while Y11 and 105.5R(r) which belong to *Y. enterocolitica* subsp. *palearctica* do not have yersiniabactin genes.

Besides having pYV virulence plasmid, highly pathogenic *Yersinia* strains also possess iron-regulated genes [63]. There have been reports stating that *Y. enterocolitica* serotype O:8 carries high pathogenicity island (HPI), which synthesizes yersiniabactin [64,65]. In order to get a better understanding of the pathogenicity of *Y. enterocolitica* subspecies, we used PathoProT to visualize the virulence gene profiles. From the heat map produced by PathoProT (part of the heat map is shown in Figure 4), we found that yersiniabactin was not present in *Y. enterocolitica* subsp. *palearctica*. The result is consistent with previous findings [62,66] and further suggests that *Y. enterocolitica* subsp. *enterocolitica* has followed a slightly different evolutionary pathway compared to subsp. *palearctica* and that yersiniabactin was acquired by *Y. enterocolitica* subsp. *enterocolitica* after it diverged from its common ancestor shared with subsp. *palearctica*.

**Figure 5:**
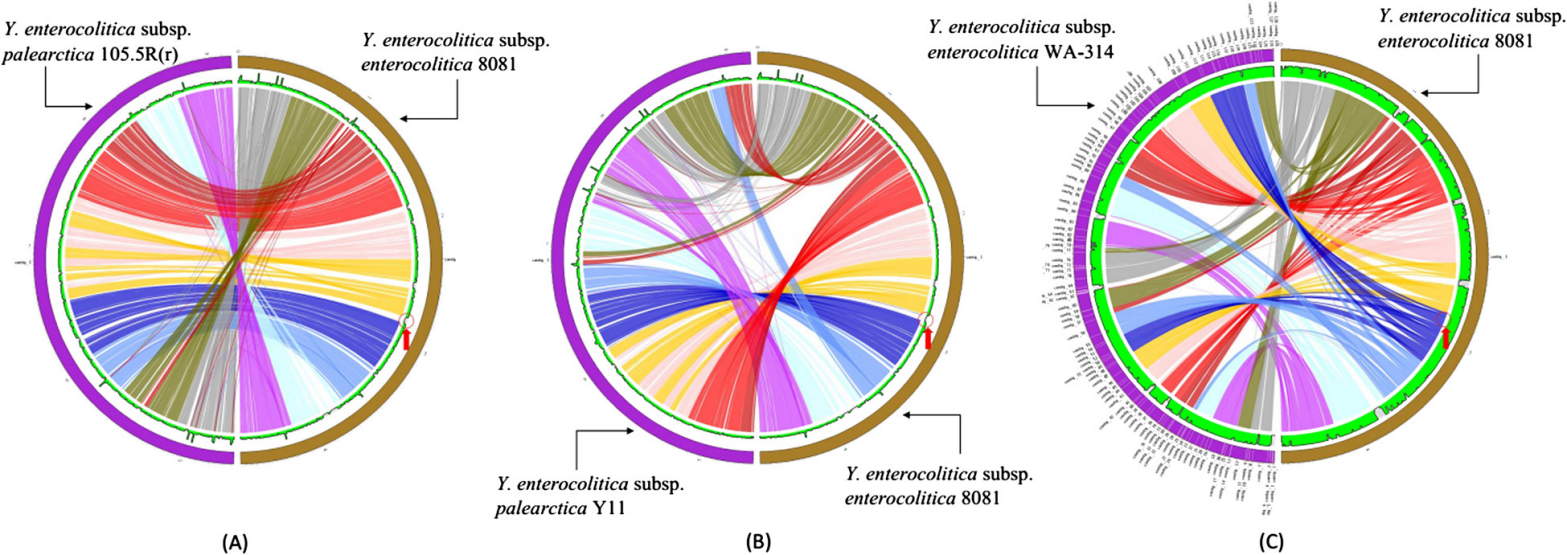
**Genome comparisons between different subspecies of Yersinia enterocolitica by using PGC tool.** PGC plots revealed yersiniabactin only present in Yersinia enterocolitica subsp. enterocolitica. **(A)** Comparison between 105.5R(r) and 8081 by using PGC showed that 105.5R(r) did not map to region of yersiniabactin in 8081. **(B)** Comparison between Y11 and 8081 by using PGC showed that Y11 did not map to region of yersiniabactin in 8081. **(C)** Comparison between WA-314 by using PGC showed that WA-314 mapped to region of yersiniabactin in 8081.

We further visualized the difference between the two subspecies of *Y. enterocolitica* by using PGC Tool with *Y. enterocolitica* 8081 as reference genome (Figure 5). Another draft genome, *Y. enterocolitica* subsp. *enterocolitica* WA-314 was included. The start and stop position of the operon for yersiniabactin in 8081 are 2817014 and 2841569 respectively (red circle region in the Figure 5). From the three figures generated by PGC (Figure 5), we found only WA-314 has region mapped to the region encoding yersiniabactin in 8081.

### Flagella genes in *Y. pestis* Angola

**Figure 6:**
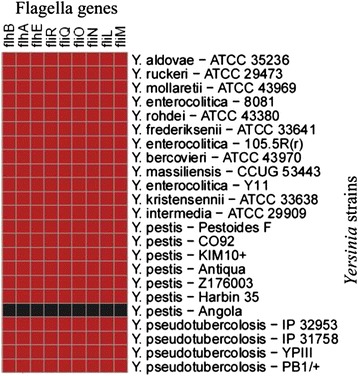
**A heat map showed the absence (black color) of nine flagella genes in Angola, but present (red color) in other selected** ***Yersinia*** **strains.**

We found 9 flagella genes missing from *Y. pestis* Angola, but present in the rest of studied strains (Figure 6). The 9 flagella genes are *flh*A, *flh*B, *flh*E, *fli*L, *fli*M, *fli*N, *fli*O, *fli*Q, and *fli*R. In order to prevent false negative results, the 9 flagella genes present in other strains but absent in Angola were used to TBLASTN against the *Y. pestis* Angola genome. Using this approach, we found that the missing flagella genes matched to the Angola genome with query coverage more than 50% (except *fli*O and *fli*L), but with identity less than 50% (Table 4).

**Table 4:** **The query sequence coverage and percentage of protein identity of nine missing flagella genes in Angola but present in other** ***Yersinia*** **strains TBLASTN against genome of Angola**

**Gene**	**Contig**	**Sequence identity (%)**	**Sequence coverage (%)**
*flh*B	Angola_contig0	36.6	97
*flh*B	Angola_contig0	29.38	97
*flh*A	Angola_contig0	47.44	88
*flh*A	Angola_contig0	46.59	88
*flh*E	Angola_contig0	40	67
*flh*E	Angola_contig0	30.56	67
*fli*R	Angola_contig0	32.13	84
*fli*R	Angola_contig0	27.27	84
*fli*R	Angola_contig0	29.52	84
*fli*Q	Angola_contig0	41.38	65
*fli*Q	Angola_contig0	44	65
*fli*O	Angola_contig0	31.25	27
*fli*N	Angola_contig0	40.78	69
*fli*N	Angola_contig0	26.56	69
*fli*L	Angola_contig0	34.21	20

In general, these flagella genes showed very low identity, suggesting that these genes might be highly mutated over evolution time.

Although *Yersinia* is non-motile, all the *Y. pestis* strains except for the strain Angola have a complete set of flagella associated genes, including flagella synthesis and flagella motor rotation [67]. There have been reports stating that mutations have occurred in the flagella and chemotaxis gene clusters [68], but sequence similarities of the *Y. pestis* flagella genes still can be found using BLAST, with the exception of *Y. pestis* Angola. We suggest that, after the emergence of other *Yersinia* strains, many mutations have taken place in the 9 flagella genes of the Angola genome and accumulated over time, which lead to low identity between Angola and other *Y. pestis*. Another possibility is that the flagella gene cluster might have been replaced in Angola with that of a different species by horizontal gene transfer.

### Future work

With the advent of next generation sequencing (NGS), we expect more *Yersinia* strains will be published. We regularly keep track of the new developments related to *Yersinia* especially release of new *Yesinia* genomes in NCBI. We are already in the process of adding new *Yersinia* genomes to the YersiniaBase and will be updating the database as and when new genomes of *Yersinia* are available. At this moment, the database is optimized for whole-genome and comparative pathogenomic analysis. However we hope to incorporate other data types as well along with new features and functionalities in future. We are working on it and we also encourage other researchers or research groups to email us at girg@um.edu.my if they wish to share their annotations, opinions, and curated data with us. We welcome the suggestions for improving this database as it will help us make this database a comprehensive one.

## Conclusions

With the advent of next-generation sequencing technologies, it is important that biological data can be stored and retrieved easily for analyses. YersiniaBase aims to provide a platform that stores genomic data and annotation details of *Yersinia* besides providing new bioinformatics tools that can assist researchers. In this paper, we have demonstrated how the tools can be used in analyses, including the identification of pathogenicity factors and genomic differences.

With the rapid advances and significant price drop in NGS technologies, we expect more *Yersinia* strains will be sequenced and published. Thus, we will continue updating the data in YersiniaBase once these genome sequences are available. In order to improve YersiniaBase, we welcome any comment on this database and the sharing of new *Yersinia* data. Users who want their data to be added into the database can do so by clicking the “Submit Annotation Update” link, at the bottom right corner of YersiniaBase. We hope that YersiniaBase will provide a comprehensive resource and analysis platform for of the *Yersinia* research community in the future.

## Availability and system requirements

YersiniaBase is available online at http://yersinia.um.edu.my. All the sequences and annotations described in this paper could be viewed and downloaded from the website. YersiniaBase is best viewed by Internet Explorer 8.x or higher, Mozilla Firefox® 10.x or higher, Safari 5.1 or higher, Chrome 18 or higher and any other equivalent browser software. If your browser is older, you may have trouble viewing many of our web site features properly. This web site is best viewed at a screen resolution of 1024 × 768 pixels or higher.

## Additional files

Additional file 1: Table S1. List of strains available in YersiniaBase and their corresponding strain name, genome status, genome size (bp), total number of contig, number of open reading frame (ORF), number of tRNA, number of rRNA and percentage of GC content. Additional file 2: Figure S1. Underlying relational database schema of YersiniaBase, which consists of four tables, with function of each table, is depicted in the figure. The relationship between tables which is defined as followed: (1) each species has one or many strains; (2) each strain has one or many open reading frames; (3) each open reading frame has only one nucleotide sequence, none or one amino acid sequence. Strain table has foreign key that links to primary key in species table; ORF table has foreign key that links to primary key in strain table; sequence table has foreign key that links to primary key in ORF table. Additional file 3: Figure S2. Overall functionalities in YersiniaBase. Additional file 4: Figure S3. (A) Green and blue link were displayed as the mapped region, because the mapped region is higher than the link threshold, while the gap is present between green and blue link because the gap is wider than the value of merge threshold (0Kbp in this case). (B) Since the gap (1Kbp) is smaller than 2Kbp (merge threshold in this case), the green and blue link beside the gap were merged into a wider link of 8Kbp (2Kbp Green Link + 1Kbp Gap + 5Kbp Blue link). Additional file 5: Figure S4. Brief description of processes taken in PGC pipeline after user submits the job to our server. Additional file 6: Figure S5. Brief description of processes taken in PathoProT pipeline after user submits the job to our server.

## Abbreviations

AJAX: Asynchronous JavaScript and XML
BLAST: Basic Local Alignment Search Tool
bp: Base pair
CDS: Coding DNA sequence
CSS: Cascading Style Sheets
EFSA: European Food Safety Authority
HPI: High pathogenicity island
HTML: HyperText Markup Language
Jbrowse: JavaScript-based genome browser
MVC: Model-view-controller
NCBI: National Center for Biotechnology Information
NGS: Next generation sequencing
ORF: Open reading frame
PathoProT: Pathogenomics Profiling Tool
PGC: Pairwise Genome Comparison tool
PHP: HyperText Preprocessor
RAST: Rapid Annotation using Subsystem Technology
RNA: Ribonucleic acid
TTSS: Type III secretion system
VFDB: Virulence factors database
WHO: World Health Organization
Yop: Yersinia outer protein
