# Supplementary material for: YersiniaBase: a genomic resource and analysis platform for comparative analysis of Yersinia
Source: BMC Bioinformatics. 2015 Jan 16;16(1):9. doi: 10.1186/s12859-014-0422-y (PMC4384384; doi:10.1186/s12859-014-0422-y)
Supplement: Additional file 1: Table S1. — List of strains available in YersiniaBase and their corresponding strain name, genome status, genome size (bp), total number of contig, number of open reading frame (ORF), number of tRNA, number of rRNA and percentage of GC content. [file 12859_2014_422_MOESM1_ESM.docx]

**Supplementary Table 1:** List of strains available in YersiniaBase and their corresponding strain name, genome status, genome size (bp), total number of contig, number of open reading frame (ORF), number of tRNA, number of rRNA and percentage of GC content.

| No | Strain name | Genome status | Genome size (bp) | Total contig | Total of ORF | Total of tRNA | Total of rRNA | GC content (%) |
| --- | --- | --- | --- | --- | --- | --- | --- | --- |
| 1 | ATCC35236 | draft | 4304926 | 210 | 4215 | 62 | 8 | 47.69 |
| 2 | ATCC43970 | draft | 4347293 | 229 | 4143 | 60 | 14 | 48.90 |
| 3 | 556/8265 | draft | 4521393 | 14 | 4258 | 61 | 5 | 45.41 |
| 4 | 647/5307 | draft | 4553105 | 18 | 4403 | 94 | 5 | 45.60 |
| 5 | 105.5R(r) | complete | 4552107 | 1 | 4387 | 72 | 22 | 47.00 |
| 6 | 8081 | complete | 4615899 | 1 | 4445 | 81 | 22 | 47.27 |
| 7 | Y11 | complete | 4553420 | 1 | 4464 | 70 | 22 | 47.01 |
| 8 | IP10393 | draft | 4463212 | 12 | 4338 | 64 | 5 | 46.96 |
| 9 | IP2222 | draft | 4753960 | 74 | 4599 | 74 | 3 | 47.13 |
| 10 | NFO | draft | 4663108 | 97 | 4548 | 74 | 11 | 47.08 |
| 11 | PhRBD_Ye1 | draft | 4367390 | 112 | 4298 | 58 | 5 | 46.94 |
| 12 | WA-314 | draft | 4524664 | 129 | 4337 | 65 | 4 | 47.18 |
| 13 | YO527 | draft | 4658995 | 20 | 4519 | 85 | 18 | 45.97 |
| 14 | ATCC33641 | draft | 4885341 | 161 | 4635 | 65 | 8 | 46.93 |
| 15 | ATCC29909 | draft | 4711317 | 123 | 4439 | 64 | 26 | 47.44 |
| 16 | ATCC33638 | draft | 5042394 | 153 | 4859 | 67 | 7 | 47.36 |
| 17 | CCUG53443 | draft | 5052395 | 22 | 4762 | 67 | 7 | 47.19 |
| 18 | ATCC43969 | draft | 4549461 | 179 | 4236 | 54 | 5 | 49.02 |
| 19 | 164 | draft | 4432709 | 1050 | 4208 | 21 | 5 | 47.83 |
| 20 | 2 | draft | 4453910 | 1057 | 4227 | 20 | 4 | 47.93 |
| 21 | 2330 | draft | 4427639 | 798 | 4264 | 21 | 3 | 47.54 |
| 22 | 2501 | draft | 5265077 | 216 | 5230 | 70 | 5 | 47.49 |
| 23 | 2504 | draft | 4475655 | 975 | 4288 | 17 | 1 | 47.57 |
| 24 | 2506 | draft | 4488493 | 820 | 4283 | 21 | 3 | 47.58 |
| 25 | 2654 | draft | 4352602 | 2718 | 4101 | 21 | 3 | 47.59 |
| 26 | 30017 | draft | 4591472 | 747 | 4434 | 21 | 4 | 47.63 |
| 27 | 34008 | draft | 4375742 | 1074 | 4112 | 21 | 4 | 47.99 |
| 28 | 34202 | draft | 4504947 | 1237 | 4314 | 21 | 6 | 47.67 |
| 29 | 351001 | draft | 4433848 | 1332 | 4178 | 21 | 5 | 47.96 |
| 30 | 42013 | draft | 4495101 | 877 | 4299 | 21 | 7 | 47.60 |
| 31 | 42082 | draft | 4496670 | 2049 | 4280 | 21 | 3 | 47.65 |
| 32 | 42091 | draft | 4495831 | 937 | 4297 | 20 | 7 | 47.62 |
| 33 | 42095 | draft | 4512584 | 1119 | 4351 | 21 | 4 | 47.61 |
| 34 | 5 | draft | 4478071 | 1008 | 4297 | 21 | 5 | 47.56 |
| 35 | 5761 | draft | 4262011 | 945 | 4053 | 21 | 5 | 47.68 |
| 36 | 620024 | draft | 4503786 | 1131 | 4291 | 22 | 0 | 47.61 |
| 37 | 7 | draft | 4539616 | 768 | 4367 | 21 | 4 | 47.54 |
| 38 | 71021 | draft | 4493411 | 1489 | 4246 | 21 | 5 | 47.74 |
| 39 | 710317 | draft | 4449879 | 1119 | 4243 | 21 | 3 | 47.72 |
| 40 | 7338 | draft | 4344510 | 1074 | 4199 | 21 | 7 | 47.50 |
| 41 | 735 | draft | 4423715 | 882 | 4298 | 20 | 1 | 47.44 |
| 42 | 780441 | draft | 4525764 | 817 | 4341 | 21 | 3 | 47.59 |
| 43 | 9 | draft | 4452201 | 1336 | 4228 | 20 | 6 | 47.72 |
| 44 | 91 | draft | 4366271 | 906 | 4178 | 21 | 4 | 47.69 |
| 45 | 945 | draft | 4226749 | 1243 | 3902 | 21 | 4 | 48.09 |
| 46 | 970754 | draft | 4348631 | 1104 | 4063 | 20 | 4 | 47.96 |
| 47 | A1956001 | draft | 4508534 | 852 | 4325 | 21 | 4 | 47.60 |
| 48 | A1973001 | draft | 4413119 | 1539 | 4253 | 21 | 3 | 47.60 |
| 49 | B42003004 | draft | 4841690 | 69 | 4797 | 63 | 28 | 47.68 |
| 50 | E1979001_PRJNA198911 | draft | 4516254 | 941 | 4343 | 20 | 3 | 47.51 |
| 51 | E1979001_PRJNA54471 | draft | 4847813 | 75 | 4745 | 60 | 27 | 47.69 |
| 52 | UG05-0454 | draft | 4838246 | 98 | 4791 | 67 | 32 | 47.73 |
| 53 | K1973002 | draft | 4720694 | 73 | 4676 | 66 | 33 | 47.63 |
| 54 | AS200901156 | draft | 4578309 | 220 | 4547 | 59 | 4 | 47.49 |
| 55 | AS200901434 | draft | 4572981 | 237 | 4533 | 58 | 4 | 47.49 |
| 56 | AS200901509 | draft | 4605070 | 263 | 4588 | 59 | 8 | 47.51 |
| 57 | AS200901539 | draft | 4572127 | 250 | 4565 | 52 | 4 | 47.49 |
| 58 | AS200902147 | draft | 4592682 | 277 | 4665 | 58 | 5 | 47.50 |
| 59 | BA200901703 | draft | 4560176 | 282 | 4546 | 55 | 2 | 47.48 |
| 60 | BA200901799 | draft | 4581111 | 199 | 4513 | 60 | 4 | 47.50 |
| 61 | BA200901990 | draft | 4527493 | 210 | 4429 | 60 | 4 | 47.55 |
| 62 | BA200902009 | draft | 4563812 | 256 | 4537 | 52 | 4 | 47.47 |
| 63 | F1991016 | draft | 4892085 | 107 | 4803 | 60 | 23 | 47.69 |
| 64 | IP275 | draft | 5139152 | 101 | 5366 | 52 | 22 | 47.93 |
| 65 | MG05-1020 | draft | 4977609 | 80 | 4940 | 68 | 26 | 47.61 |
| 66 | C1975003 | draft | 4365481 | 1452 | 4074 | 21 | 3 | 48.24 |
| 67 | C1976001 | draft | 4546693 | 979 | 4402 | 20 | 0 | 47.46 |
| 68 | C1989001 | draft | 4588735 | 934 | 4442 | 19 | 3 | 47.57 |
| 69 | CA88-4125 | draft | 4650262 | 7 | 4505 | 67 | 20 | 47.63 |
| 70 | CMCC02041 | draft | 4489186 | 1281 | 4295 | 21 | 8 | 47.59 |
| 71 | CMCC03001 | draft | 4366616 | 1082 | 4155 | 21 | 3 | 47.61 |
| 72 | CMCC05009 | draft | 4501730 | 1501 | 4253 | 23 | 6 | 47.73 |
| 73 | CMCC05013 | draft | 4395097 | 1360 | 4112 | 21 | 3 | 47.84 |
| 74 | CMCC10012 | draft | 4435712 | 1001 | 4221 | 21 | 3 | 47.74 |
| 75 | CMCC104003 | draft | 4494768 | 1387 | 4254 | 20 | 9 | 47.68 |
| 76 | CMCC106002 | draft | 4424589 | 1176 | 4151 | 21 | 4 | 47.81 |
| 77 | CMCC107004 | draft | 4455583 | 2814 | 4221 | 17 | 0 | 47.47 |
| 78 | CMCC11001 | draft | 4423348 | 1100 | 4181 | 21 | 3 | 47.92 |
| 79 | CMCC114001 | draft | 4231517 | 1664 | 3846 | 23 | 4 | 48.23 |
| 80 | CMCC12003 | draft | 4341435 | 1045 | 4137 | 21 | 4 | 47.77 |
| 81 | CMCC125002 | draft | 4518010 | 790 | 4339 | 21 | 4 | 47.56 |
| 82 | CMCC18019 | draft | 4495992 | 1478 | 4295 | 21 | 4 | 47.62 |
| 83 | CMCC21106 | draft | 4251990 | 1387 | 3971 | 21 | 3 | 47.99 |
| 84 | CMCC27002 | draft | 4282597 | 1451 | 3970 | 21 | 4 | 48.13 |
| 85 | CMCC31004 | draft | 4413744 | 1229 | 4115 | 21 | 3 | 48.13 |
| 86 | CMCC347001 | draft | 4514951 | 1207 | 4311 | 19 | 4 | 47.65 |
| 87 | CMCC348002 | draft | 4571405 | 846 | 4424 | 21 | 3 | 47.55 |
| 88 | CMCC38001 | draft | 4483258 | 1030 | 4305 | 21 | 5 | 47.60 |
| 89 | CMCC42007 | draft | 4440824 | 1040 | 4217 | 21 | 3 | 47.85 |
| 90 | CMCC43032 | draft | 4512744 | 1049 | 4310 | 20 | 2 | 47.57 |
| 91 | CMCC49003 | draft | 4534555 | 795 | 4347 | 21 | 4 | 47.56 |
| 92 | CMCC51020 | draft | 4420992 | 1118 | 4152 | 21 | 4 | 47.92 |
| 93 | CMCC64001 | draft | 4360492 | 955 | 4150 | 21 | 5 | 47.72 |
| 94 | CMCC640047 | draft | 4305752 | 1490 | 3964 | 21 | 3 | 48.16 |
| 95 | CMCC67001 | draft | 4340671 | 1099 | 4116 | 20 | 5 | 47.79 |
| 96 | CMCC71001 | draft | 4254189 | 1744 | 3863 | 21 | 4 | 48.18 |
| 97 | CMCC8211 | draft | 4417626 | 797 | 4214 | 21 | 3 | 47.54 |
| 98 | CMCC84033 | draft | 4459052 | 1055 | 4266 | 18 | 2 | 47.70 |
| 99 | CMCC84038 | draft | 4438544 | 2446 | 4183 | 18 | 3 | 47.58 |
| 100 | CMCC84046 | draft | 4517175 | 1137 | 4382 | 18 | 0 | 47.46 |
| 101 | CMCC87001 | draft | 4510364 | 870 | 4325 | 23 | 3 | 47.59 |
| 102 | CMCC90027 | draft | 4358986 | 1102 | 4157 | 21 | 3 | 47.79 |
| 103 | CMCC91090 | draft | 4418766 | 987 | 4212 | 21 | 5 | 47.68 |
| 104 | CMCC92004 | draft | 4264825 | 1461 | 3964 | 21 | 3 | 47.95 |
| 105 | CMCC92010 | draft | 4384955 | 1216 | 4200 | 21 | 7 | 47.65 |
| 106 | CMCC93014 | draft | 4507171 | 993 | 4350 | 20 | 2 | 47.51 |
| 107 | CMCC95001 | draft | 4337306 | 1361 | 4033 | 21 | 4 | 48.07 |
| 108 | CMCC96001 | draft | 4517589 | 907 | 4312 | 21 | 3 | 47.60 |
| 109 | CMCC96007 | draft | 4303407 | 1563 | 3994 | 20 | 11 | 48.15 |
| 110 | CMCC99103 | draft | 4409843 | 1262 | 4170 | 21 | 5 | 47.96 |
| 111 | CMCCK100001 | draft | 4520971 | 1406 | 4375 | 20 | 0 | 47.43 |
| 112 | CMCCK110001 | draft | 4305250 | 1385 | 3986 | 23 | 3 | 48.13 |
| 113 | CMCCN010025 | draft | 4437913 | 1016 | 4221 | 21 | 4 | 47.79 |
| 114 | 91001 | complete | 4595065 | 1 | 4486 | 72 | 22 | 47.65 |
| 115 | A1122 | complete | 4553770 | 1 | 4442 | 69 | 18 | 47.58 |
| 116 | Angola | complete | 4504254 | 1 | 4416 | 70 | 22 | 47.60 |
| 117 | Antiqua | complete | 4702289 | 1 | 4593 | 68 | 22 | 47.70 |
| 118 | CO92 | complete | 4653728 | 1 | 4541 | 70 | 19 | 47.64 |
| 119 | D106004 | complete | 4640720 | 1 | 4635 | 70 | 19 | 47.63 |
| 120 | D182038 | complete | 4626944 | 1 | 4652 | 73 | 19 | 47.63 |
| 121 | Harbin35 | complete | 4532063 | 1 | 4474 | 72 | 22 | 47.59 |
| 122 | KIM10+ | complete | 4600755 | 1 | 4498 | 73 | 22 | 47.64 |
| 123 | Nepal516 | complete | 4534590 | 1 | 4444 | 72 | 22 | 47.58 |
| 124 | Pestoides F | complete | 4517345 | 1 | 4436 | 72 | 22 | 47.64 |
| 125 | Z176003 | complete | 4553586 | 1 | 4481 | 68 | 19 | 47.68 |
| 126 | D1964001 | draft | 4389464 | 1012 | 4214 | 21 | 8 | 47.50 |
| 127 | D1964002 | draft | 4431847 | 1555 | 4202 | 19 | 3 | 47.58 |
| 128 | D1982001 | draft | 4410882 | 896 | 4296 | 20 | 0 | 47.39 |
| 129 | D1991004 | draft | 4506081 | 1270 | 4345 | 21 | 3 | 47.54 |
| 130 | E1977001 | draft | 4385578 | 1183 | 4136 | 20 | 5 | 47.99 |
| 131 | EV76 | draft | 4274282 | 1172 | 4042 | 23 | 3 | 47.88 |
| 132 | F1946001 | draft | 4463713 | 1383 | 4263 | 23 | 3 | 47.50 |
| 133 | F1954001 | draft | 4521000 | 1058 | 4348 | 17 | 2 | 47.46 |
| 134 | F1984001 | draft | 4326553 | 1467 | 4051 | 24 | 4 | 48.10 |
| 135 | FV-1 | draft | 4472646 | 400 | 4584 | 5 | 0 | 47.59 |
| 136 | G1996006 | draft | 4542684 | 842 | 4366 | 21 | 3 | 47.65 |
| 137 | G1996010 | draft | 4552418 | 772 | 4386 | 21 | 3 | 47.61 |
| 138 | G8786 | draft | 4325798 | 829 | 4129 | 20 | 3 | 47.87 |
| 139 | H1958004 | draft | 4195425 | 1339 | 3910 | 22 | 4 | 48.08 |
| 140 | H1959004 | draft | 4470270 | 946 | 4261 | 21 | 5 | 47.75 |
| 141 | I160001 | draft | 4399336 | 1222 | 4186 | 21 | 6 | 47.62 |
| 142 | I1969003 | draft | 4512122 | 1213 | 4340 | 20 | 8 | 47.60 |
| 143 | I1970005 | draft | 4429836 | 1199 | 4174 | 21 | 3 | 47.89 |
| 144 | I1991001 | draft | 4335804 | 1191 | 4048 | 21 | 4 | 48.02 |
| 145 | I1994006 | draft | 4498336 | 1247 | 4292 | 21 | 7 | 47.66 |
| 146 | I2001001 | draft | 4509469 | 1526 | 4397 | 19 | 0 | 47.46 |
| 147 | INS | draft | 4651700 | 18 | 4531 | 70 | 19 | 47.63 |
| 148 | J1963002 | draft | 4434765 | 1119 | 4189 | 21 | 4 | 47.83 |
| 149 | J1978002 | draft | 4532297 | 1098 | 4370 | 18 | 1 | 47.47 |
| 150 | K11973002 | draft | 4383307 | 910 | 4187 | 21 | 4 | 47.63 |
| 151 | K21985002 | draft | 4399142 | 1264 | 4219 | 22 | 4 | 47.65 |
| 152 | M0000002 | draft | 4441674 | 1135 | 4172 | 21 | 4 | 47.78 |
| 153 | MGJZ11 | draft | 4516720 | 859 | 4351 | 21 | 6 | 47.60 |
| 154 | MGJZ12 | draft | 4438965 | 1203 | 4231 | 22 | 6 | 47.91 |
| 155 | MGJZ3 | draft | 4453871 | 1187 | 4288 | 17 | 0 | 47.41 |
| 156 | MGJZ6 | draft | 4368072 | 1016 | 4172 | 21 | 9 | 47.87 |
| 157 | MGJZ7 | draft | 4365909 | 1325 | 4143 | 21 | 7 | 47.91 |
| 158 | MGJZ9 | draft | 4474930 | 1082 | 4289 | 21 | 5 | 47.90 |
| 159 | PY-01 | draft | 4376444 | 534 | 4385 | 62 | 4 | 47.47 |
| 160 | PY-02 | draft | 4339287 | 539 | 4379 | 61 | 5 | 47.39 |
| 161 | PY-03 | draft | 3953166 | 626 | 3946 | 50 | 6 | 47.66 |
| 162 | PY-04 | draft | 4588154 | 514 | 4578 | 64 | 3 | 47.60 |
| 163 | PY-05 | draft | 4685275 | 479 | 4692 | 63 | 4 | 47.48 |
| 164 | PY-06 | draft | 4765471 | 524 | 4686 | 63 | 4 | 47.55 |
| 165 | PY-07 | draft | 4764013 | 471 | 4771 | 63 | 4 | 47.54 |
| 166 | PY-08 | draft | 4736966 | 508 | 4771 | 63 | 4 | 47.51 |
| 167 | PY-09 | draft | 4431218 | 521 | 4462 | 61 | 4 | 47.47 |
| 168 | PY-10 | draft | 4552981 | 521 | 4580 | 63 | 4 | 47.46 |
| 169 | PY-100 | draft | 4631765 | 491 | 4640 | 63 | 5 | 47.55 |
| 170 | PY-101 | draft | 4732273 | 500 | 4697 | 65 | 4 | 47.56 |
| 171 | PY-102 | draft | 4501667 | 519 | 4502 | 62 | 5 | 47.49 |
| 172 | PY-103 | draft | 3549825 | 717 | 3488 | 42 | 5 | 47.77 |
| 173 | PY-11 | draft | 4325459 | 558 | 4357 | 59 | 1 | 47.46 |
| 174 | PY-113 | draft | 4373787 | 550 | 4362 | 58 | 3 | 47.59 |
| 175 | PY-12 | draft | 4827600 | 491 | 4846 | 68 | 3 | 47.42 |
| 176 | PY-13 | draft | 4347671 | 532 | 4368 | 59 | 4 | 47.43 |
| 177 | PY-14 | draft | 4017796 | 601 | 3944 | 52 | 5 | 47.80 |
| 178 | PY-15 | draft | 4739423 | 501 | 4729 | 63 | 3 | 47.50 |
| 179 | PY-16 | draft | 4687256 | 481 | 4679 | 62 | 6 | 47.48 |
| 180 | PY-19 | draft | 4363686 | 517 | 4314 | 59 | 6 | 47.44 |
| 181 | PY-25 | draft | 4608038 | 516 | 4634 | 63 | 4 | 47.39 |
| 182 | PY-29 | draft | 4758035 | 428 | 4761 | 62 | 4 | 47.53 |
| 183 | PY-32 | draft | 4627734 | 538 | 4669 | 65 | 4 | 47.45 |
| 184 | PY-34 | draft | 4646453 | 507 | 4642 | 63 | 5 | 47.51 |
| 185 | PY-36 | draft | 4380242 | 546 | 4435 | 64 | 3 | 47.48 |
| 186 | PY-42 | draft | 4667682 | 512 | 4673 | 62 | 3 | 47.53 |
| 187 | PY-45 | draft | 4754544 | 471 | 4716 | 64 | 4 | 47.53 |
| 188 | PY-46 | draft | 4674925 | 460 | 4692 | 62 | 4 | 47.49 |
| 189 | PY-47 | draft | 4502246 | 517 | 4497 | 63 | 4 | 47.47 |
| 190 | PY-48 | draft | 4326806 | 542 | 4359 | 61 | 10 | 47.50 |
| 191 | PY-52 | draft | 4026062 | 599 | 3948 | 46 | 3 | 47.64 |
| 192 | PY-53 | draft | 4237808 | 575 | 4235 | 60 | 5 | 47.54 |
| 193 | PY-54 | draft | 3696678 | 1089 | 3494 | 35 | 3 | 47.71 |
| 194 | PY-55 | draft | 4753010 | 471 | 4752 | 64 | 4 | 47.53 |
| 195 | PY-56 | draft | 4302693 | 558 | 4340 | 57 | 3 | 47.52 |
| 196 | PY-58 | draft | 3499949 | 667 | 3420 | 45 | 5 | 47.78 |
| 197 | PY-59 | draft | 4414895 | 551 | 4452 | 56 | 3 | 47.51 |
| 198 | PY-60 | draft | 4636135 | 510 | 4649 | 63 | 3 | 47.49 |
| 199 | PY-61 | draft | 4689471 | 505 | 4690 | 62 | 3 | 47.54 |
| 200 | PY-63 | draft | 4661011 | 512 | 4627 | 63 | 4 | 47.52 |
| 201 | PY-64 | draft | 3431173 | 737 | 3405 | 40 | 3 | 47.52 |
| 202 | PY-65 | draft | 4641789 | 498 | 4660 | 62 | 4 | 47.47 |
| 203 | PY-66 | draft | 3598647 | 1065 | 3498 | 42 | 7 | 47.88 |
| 204 | PY-71 | draft | 4498491 | 477 | 4497 | 61 | 3 | 47.48 |
| 205 | PY-72 | draft | 4283224 | 522 | 4248 | 59 | 5 | 47.42 |
| 206 | PY-76 | draft | 4498987 | 462 | 4523 | 62 | 3 | 47.48 |
| 207 | PY-88 | draft | 4185691 | 591 | 4219 | 54 | 3 | 47.46 |
| 208 | PY-89 | draft | 4014866 | 602 | 3947 | 54 | 1 | 47.54 |
| 209 | PY-90 | draft | 4662510 | 506 | 4657 | 63 | 3 | 47.51 |
| 210 | PY-91 | draft | 4460984 | 521 | 4413 | 60 | 4 | 47.41 |
| 211 | PY-92 | draft | 4518071 | 522 | 4537 | 63 | 4 | 47.50 |
| 212 | PY-93 | draft | 4770670 | 487 | 4804 | 64 | 3 | 47.43 |
| 213 | PY-94 | draft | 4362113 | 542 | 4416 | 60 | 1 | 47.48 |
| 214 | PY-95 | draft | 4687267 | 456 | 4696 | 61 | 4 | 47.46 |
| 215 | PY-96 | draft | 4279809 | 540 | 4278 | 58 | 3 | 47.53 |
| 216 | PY-98 | draft | 4753558 | 510 | 4741 | 63 | 3 | 47.50 |
| 217 | PY-99 | draft | 4605671 | 509 | 4617 | 63 | 4 | 47.51 |
| 218 | SHAN11 | draft | 4384465 | 1173 | 4134 | 21 | 4 | 47.95 |
| 219 | SHAN12 | draft | 4371391 | 1362 | 4106 | 22 | 3 | 48.02 |
| 220 | YN1065 | draft | 4472301 | 1395 | 4207 | 20 | 6 | 47.69 |
| 221 | YN1683 | draft | 4302115 | 1897 | 3983 | 19 | 3 | 47.86 |
| 222 | YN2179 | draft | 4307368 | 1605 | 3984 | 22 | 4 | 48.16 |
| 223 | YN2551 | draft | 4524315 | 880 | 4358 | 22 | 3 | 47.54 |
| 224 | YN2588 | draft | 4307940 | 1301 | 4038 | 23 | 4 | 48.05 |
| 225 | YN472 | draft | 4525667 | 1105 | 4377 | 17 | 0 | 47.46 |
| 226 | YN663 | draft | 3516955 | 1035 | 3282 | 21 | 2 | 48.08 |
| 227 | IP31758 | complete | 4723306 | 1 | 4546 | 86 | 22 | 47.54 |
| 228 | IP32953 | complete | 4744671 | 1 | 4606 | 85 | 22 | 47.61 |
| 229 | PB1+ | complete | 4695619 | 1 | 4557 | 83 | 22 | 47.53 |
| 230 | YPIII | complete | 4689441 | 1 | 4596 | 84 | 22 | 47.53 |
| 231 | ATCC43380 | draft | 4317478 | 141 | 4076 | 51 | 6 | 46.97 |
| 232 | ATCC29473 | draft | 3739990 | 174 | 3550 | 47 | 6 | 47.42 |
