# Supplementary figures and images for: YersiniaBase: a genomic resource and analysis platform for comparative analysis of Yersinia
Source: BMC Bioinformatics. 2015 Jan 16;16(1):9. doi: 10.1186/s12859-014-0422-y (PMC4384384; doi:10.1186/s12859-014-0422-y)

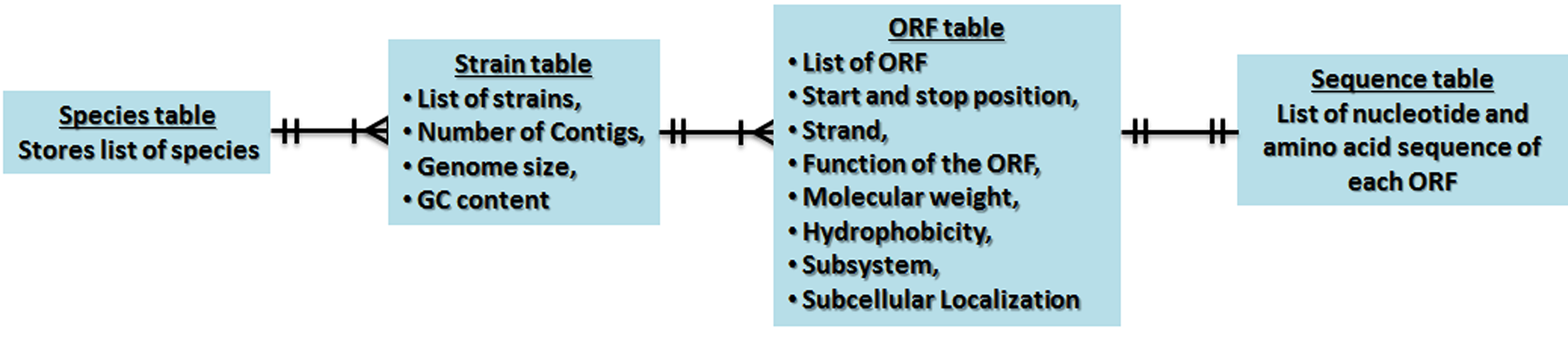

Supplement: Additional file 2: Figure S1. — Underlying relational database schema of YersiniaBase, which consists of four tables, with function of each table, is depicted in the figure. The relationship between tables which is defined as followed: (1) each species has one or many strains; (2) each strain has one or many open reading frames; (3) each open reading frame has only one nucleotide sequence, none or one amino acid sequence. Strain table has foreign key that links to primary key in species table; ORF table has foreign key that links to primary key in strain table; sequence table has foreign key that links to primary key in ORF table. [file 12859_2014_422_MOESM2_ESM.tiff]

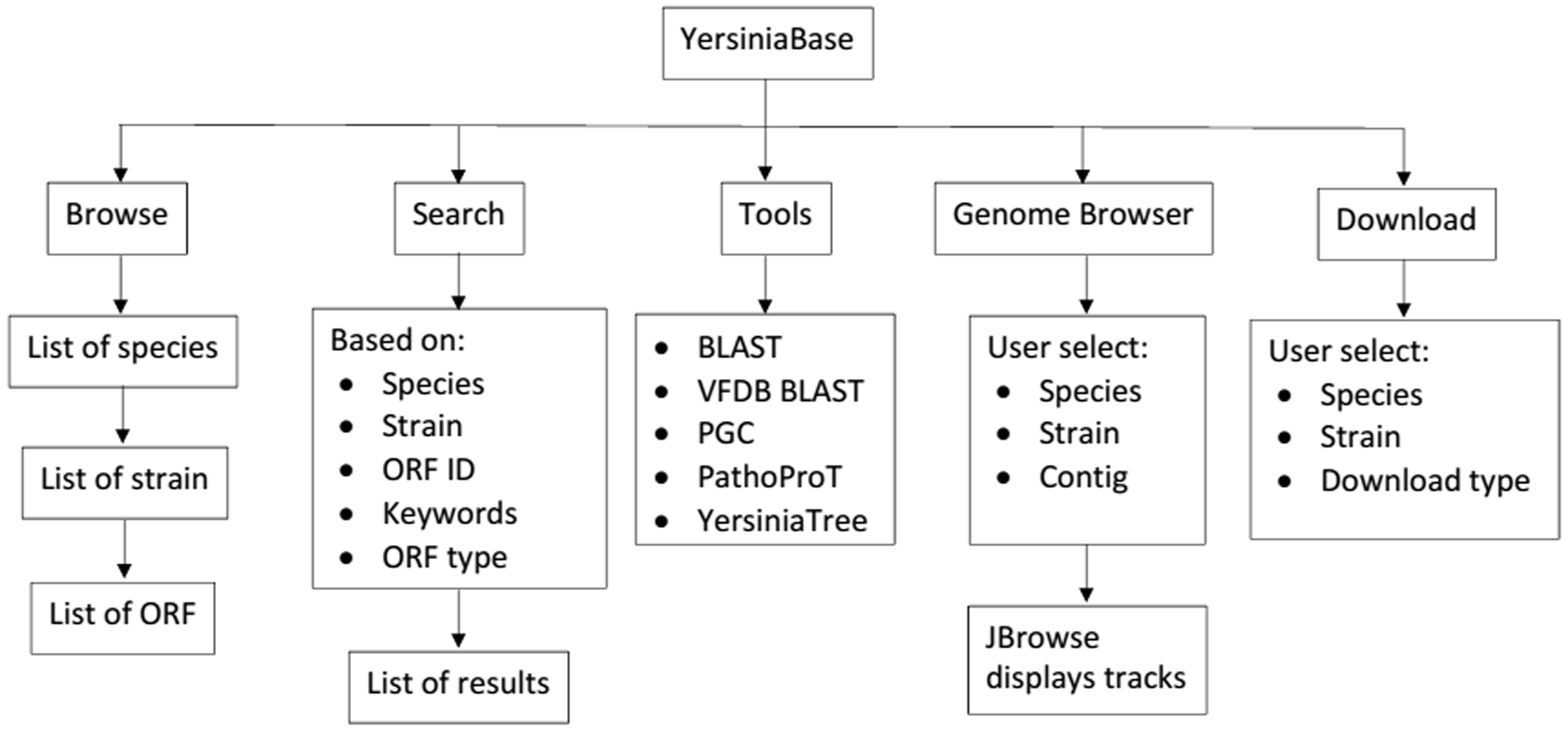

Supplement: Additional file 3: Figure S2. — Overall functionalities in YersiniaBase. [file 12859_2014_422_MOESM3_ESM.tiff]

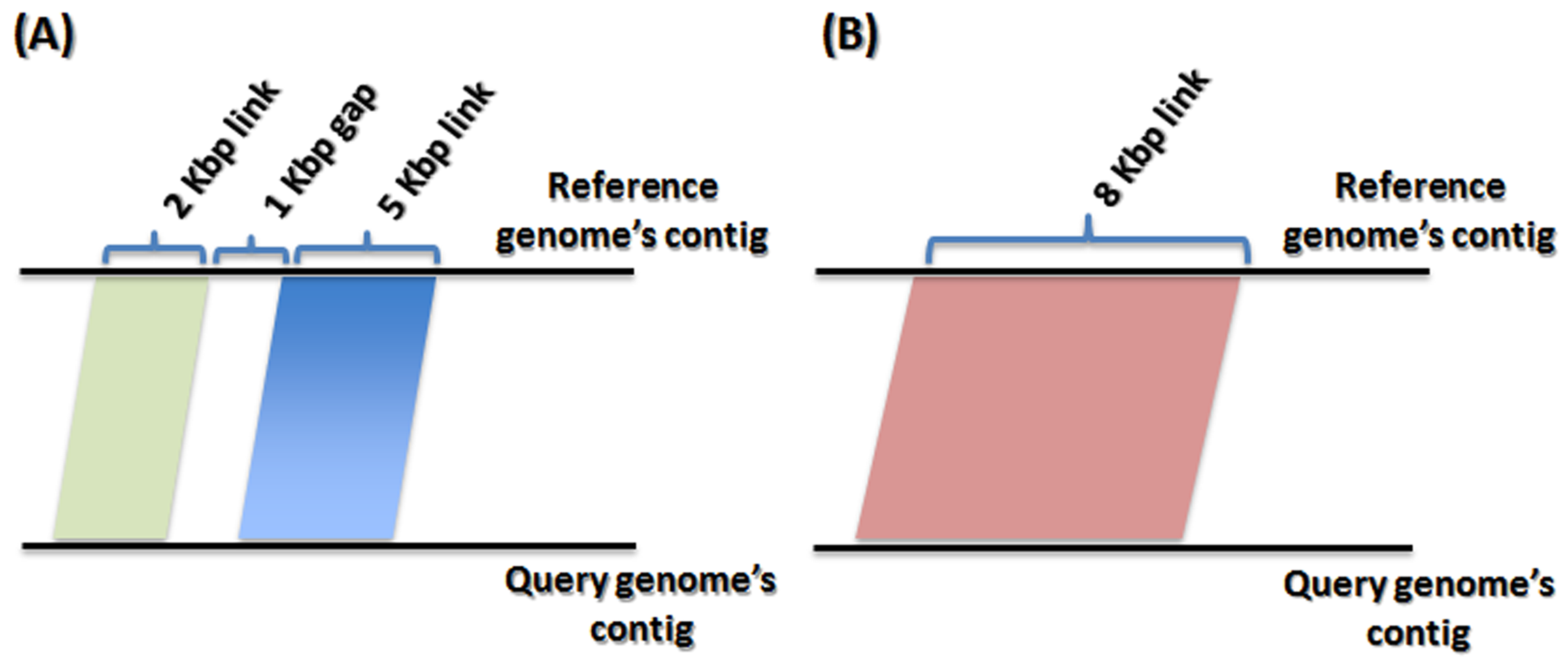

Supplement: Additional file 4: Figure S3. — (A) Green and blue link were displayed as the mapped region, because the mapped region is higher than the link threshold, while the gap is present between green and blue link because the gap is wider than the value of merge threshold (0Kbp in this case). (B) Since the gap (1Kbp) is smaller than 2Kbp (merge threshold in this case), the green and blue link beside the gap were merged into a wider link of 8Kbp (2Kbp Green Link + 1Kbp Gap + 5Kbp Blue link). [file 12859_2014_422_MOESM4_ESM.tiff]
