# Supplementary material for: YersiniaBase: a genomic resource and analysis platform for comparative analysis of Yersinia
Source: BMC Bioinformatics. 2015 Jan 16;16(1):9. doi: 10.1186/s12859-014-0422-y (PMC4384384; doi:10.1186/s12859-014-0422-y)
Supplement: Additional file 5: Figure S4. — Brief description of processes taken in PGC pipeline after user submits the job to our server. [file 12859_2014_422_MOESM5_ESM.pdf]

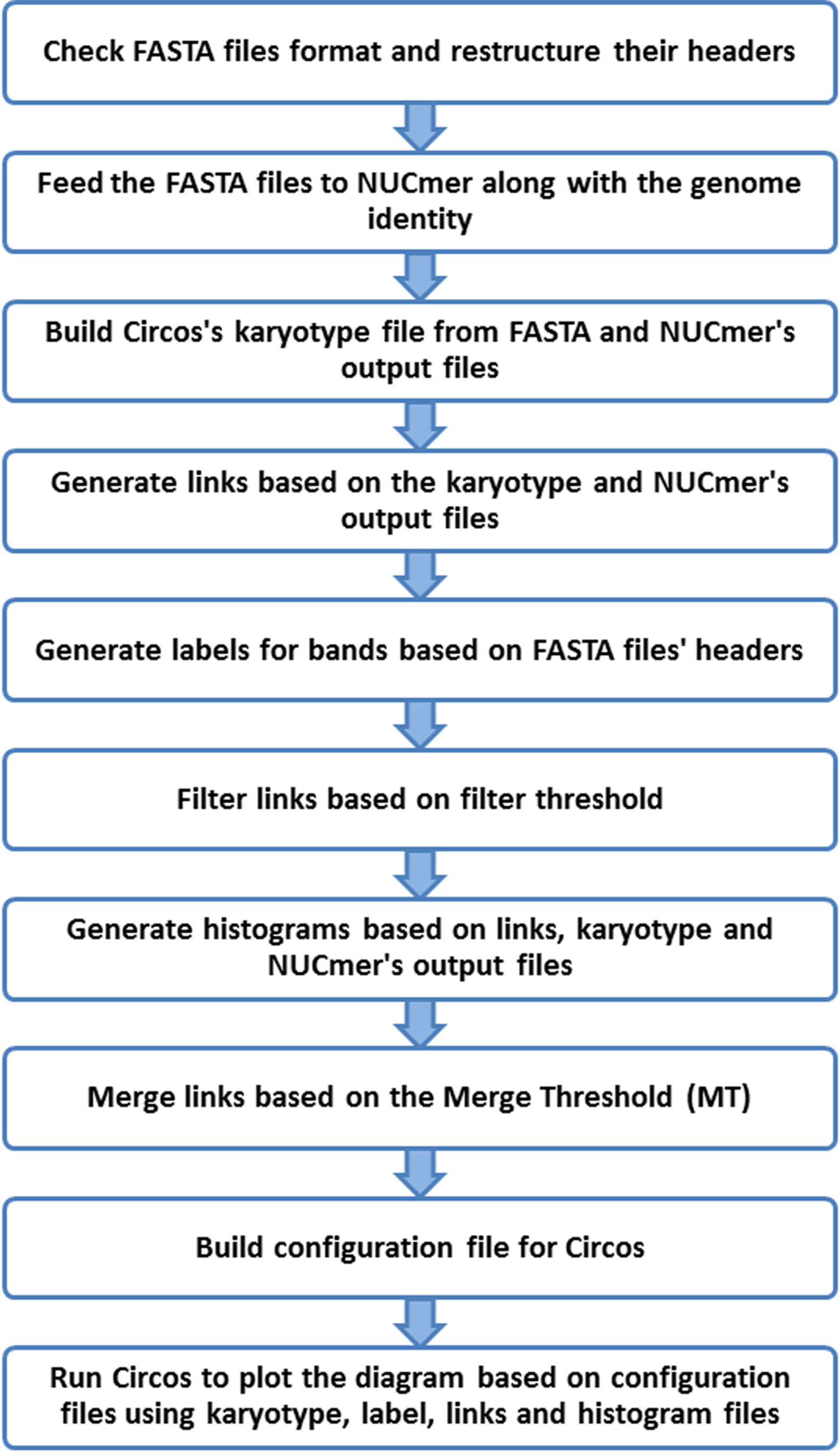
Check FASTA files format and restructure their headers
Feed the FASTA files to NUCmer along with the genome identity
Build Circos's karyotype file from FASTA and NUCmer's output files
Generate links based on the karyotype and NUCmer's output files
Generate labels for bands based on FASTA files' headers
Filter links based on filter threshold
Generate histograms based on links, karyotype and NUCmer's output files
Merge links based on the Merge Threshold (MT)
Build configuration file for Circos
Run Circos to plot the diagram based on configuration files using karyotype, label, links and histogram files
