# Supplementary material for: YersiniaBase: a genomic resource and analysis platform for comparative analysis of Yersinia
Source: BMC Bioinformatics. 2015 Jan 16;16(1):9. doi: 10.1186/s12859-014-0422-y (PMC4384384; doi:10.1186/s12859-014-0422-y)
Supplement: Additional file 6: Figure S5. — Brief description of processes taken in PathoProT pipeline after user submits the job to our server. [file 12859_2014_422_MOESM6_ESM.pdf]

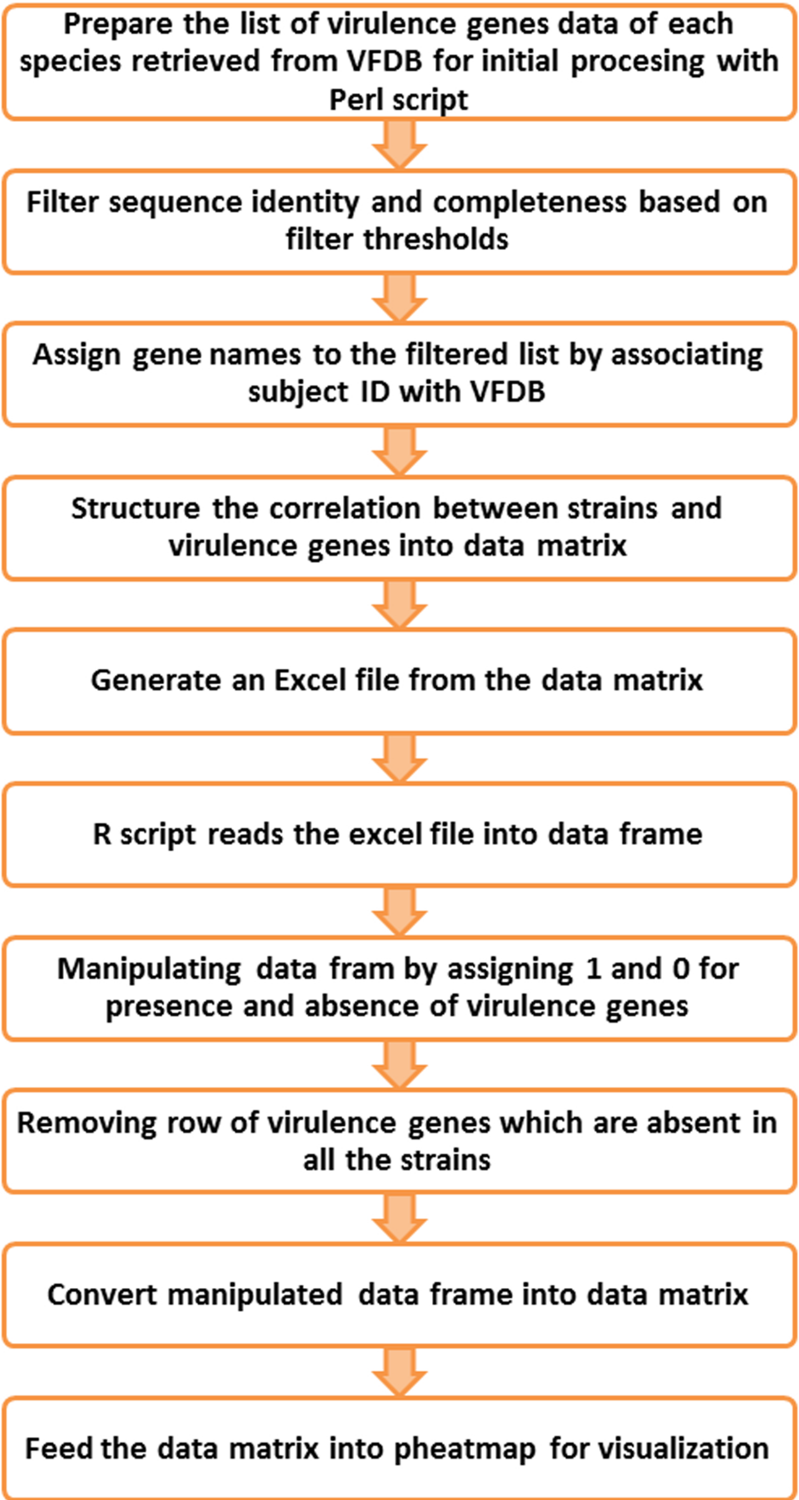
Prepare the list of virulence genes data of each species retrieved from VFDB for initial procesing with Perl script
Filter sequence identity and completeness based on filter thresholds
Assign gene names to the filtered list by associating subject ID with VFDB
Structure the correlation between strains and virulence genes into data matrix
Generate an Excel file from the data matrix
R script reads the excel file into data frame
Manipulating data fram by assigning 1 and 0 for presence and absence of virulence genes
Removing row of virulence genes which are absent in all the strains
Convert manipulated data frame into data matrix
Feed the data matrix into pheatmap for visualization
